# Phenotyping placental oxygenation in *Lgals1* deficient mice using ^19^F MRI

**DOI:** 10.1038/s41598-020-80408-9

**Published:** 2021-01-22

**Authors:** Philipp Boehm-Sturm, Susanne Mueller, Nancy Freitag, Sophia Borowski, Marco Foddis, Stefan P. Koch, Sebastian Temme, Ulrich Flögel, Sandra M. Blois

**Affiliations:** 1 Department of Experimental Neurology and Center for Stroke Research Berlin, Charité-Universitätsmedizin Berlin, corporate member of Freie Universität Berlin, Humboldt-Universität zu Berlin, and Berlin Institute of Health, Berlin, Germany; 2 NeuroCure Cluster of Excellence and Charité Core Facility 7T Experimental MRIs, Charité-Universitätsmedizin Berlin, Berlin, Germany; 3 Department for Obstetrics and Fetal Medicine, University Medical Center Hamburg-Eppendorf, Hamburg, Germany; 4 Experimental and Clinical Research Center, a Cooperation Between the Max-Delbrück Center for Molecular Medicine in the Helmholtz Association, Charité-Universitätsmedizin Berlin, Berlin, Germany; 5 Division of General Internal and Psychosomatic Medicine, Charité-Universitätsmedizin Berlin, Berlin, Germany; 6 Department of Molecular Cardiology, Heinrich-Heine-University of Düsseldorf, Düsseldorf, Germany

**Keywords:** Reproductive disorders, Nanoparticles

## Abstract

Placental hypoperfusion and hypoxia are key drivers in complications during fetal development such as fetal growth restriction and preeclampsia. In order to study the mechanisms of disease in mouse models, the development of quantitative biomarkers of placental hypoxia is a prerequisite. The goal of this exploratory study was to establish a technique to noninvasively characterize placental partial pressure of oxygen (PO_2_) in vivo in the *Lgals1* (lectin, galactoside-binding, soluble, 1) deficient mouse model of preeclampsia using fluorine magnetic resonance imaging. We hypothesized a decrease in placental oxygenation in knockout mice. Wildtype and knockout animals received fluorescently labeled perfluoro-5-crown-15-ether nanoemulsion i.v. on day E14-15 during pregnancy. Placental PO_2_ was assessed via calibrated ^19^F MRI saturation recovery T_1_ mapping. A gas challenge with varying levels of oxygen in breathing air (30%, 60% and 100% O_2_) was used to validate that changes in oxygenation can be detected in freely breathing, anesthetized animals. At the end of the experiment, fluorophore-coupled lectin was injected i.v. to label the vasculature for histology. Differences in PO_2_ between breathing conditions and genotype were statistically analyzed with linear mixed-effects modeling. As expected, a significant increase in PO_2_ with increasing oxygen in breathing air was found. PO_2_ in *Lgals1* knockout animals was decreased but this effect was only present at 30% oxygen in breathing air, not at 60% and 100%. Histological examinations showed crossing of the perfluorocarbon nanoemulsion to the fetal blood pool but the dominating contribution of ^19^F MR signal is estimated at > 70% from maternal plasma based on volume fraction measurements of previous studies. These results show for the first time that ^19^F MRI can characterize oxygenation in mouse models of placental malfunction.

## Introduction

Noninvasive examination of the placenta is of utmost importance in order to study pregnancy complications. In humans, ultrasound imaging is standard procedure to investigate the fetal and placental health without conferring risks to the mother or the unborn child. Ultrasonic examination allows the determination of fetal weight and size, inspection of fetal organs, detection of certain malformations or developmental disorders and measurements of motion sequences. Doppler ultrasonography allows studies of blood circulation in the fetus, placenta and uterus^1^. Magnetic resonance imaging (MRI) is currently indicated in certain maternal conditions such as appendicitis, pulmonary embolism, renal colic, and trauma and provides excellent prospects for translational research^2–6^. MRI of hypoperfusion has been shown to be a marker of intrauterine growth restriction^7^ and recent advances in pulse sequence design may enable a broader dissemination of placental perfusion MRI for clinical studies and diagnosis^8^. MRI was also successfully applied to investigate pregnancy in rodent models including dynamic contrast-enhanced MRI^9–11^, arterial spin labeling of placental perfusion^12^, diffusion MR of placental compartmental flow dynamics^13^, or blood/tissue oxygen level dependent (BOLD/TOLD) MRI of hemoglobin oxygenation^14^. A major advantage of small animal MRI is the possibility to follow dynamic processes over time in repetitive sessions, which is not possible in histological examinations.

The partial pressure of oxygen (PO_2_) is the most relevant parameter for the body’s oxygen sensing systems^15^ and all ^1^H MR methods (BOLD/TOLD/perfusion MRI) provide only surrogate markers of PO_2_. More direct techniques such as polarographic electrodes, fiberoptic probes or optical imaging suffer from limited penetration depth and are invasive for deeper tissue^16^. A particularly promising technique for preclinical studies of placental blood oxygenation saturation (SO_2_) is optoacoustics but it cannot directly assess PO_2_^17–19^. Therefore, there is a strong need for other spatially-resolved techniques to measure the absolute partial pressure of oxygen of the placenta in vivo. Since the fluorine (^19^F) longitudinal relaxation rate R_1_ = 1/T_1_of several perfluorocarbons (PFCs) correlates with PO_2_, ^19^F MRI has been used to study deep tissue oxygenation in animal models of disease including tumors, kidney injury or cerebral hypoperfusion^20–25^. The investigation of dynamic vascular changes and placental perfusion and oxygenation by MRI is a promising opportunity to explore angiogenesis-related pregnancy disease.

Pregnancy acts as a cardiac stress model inducing in some cases adverse cardiac events in healthy women without any previously known cardiovascular disease^26–28^. As a consequence of such cardiac stress 5–7% of all pregnancies develop preeclampsia which is associated with hypertension after the 20th week of gestation (with or without proteinuria), in conjunction with fetal growth restriction, maternal endothelial dysfunction and chronic immune inflammation^29–31^. Preeclampsia imposes a maternal increased risk of cardiovascular disease death later in life, independent of other measured risk factors. Moreover, preeclampsia is a major underlying cause of late fetal and early neonatal mortality^29,30^. A poor utero-placenta circulation secondary to inadequate remodeling compromises nutrition and oxygenation of the fetus and is associated with fetal growth restriction^29,30^.

Among the immunoregulatory and angiogenic factors, galectin-1 (gal-1), a member of a family of carbohydrate-binding proteins, has been shown to modulate several processes associated with placentation, promotion of maternal tolerance toward fetal antigens and regulation of decidual vascular expansion during the pre-placentation period^32–34^. Blocking gal-1–mediated angiogenesis with anginex, a 33-mer cytokine-like artificial β-peptide, results in a spontaneous preeclampsia-like syndrome in mice, mainly by deregulating processes associated with good placentation and maternal spiral artery remodeling^35^. Consistent with these findings, using gal-1 deficient dams we demonstrated the development of a preeclamptic-like phenotype in which mice developed gestational hypertension, proteinuria, smaller litters and progressive glomerulosclerosis. In addition to this, gal-1 deficient mice also demonstrated endothelial dysfunction, abnormal maternal decidual arteries, increased vascular resistance in the uterine arteries and poor placental development^35^.

The goal of this exploratory study was to investigate the potential of ^19^F MRI to measure placental oxygenation after i.v. injection of a Perfluoro-15-crown-5-ether (PFCE) emulsion in the gal-1 deficient *Lgals1*^*−/−*^ knockout mouse, hypothesizing a PO_2_ deficit in this model of preeclampsia.

## Results

### Method validation by gas challenge

**Figure 1:**
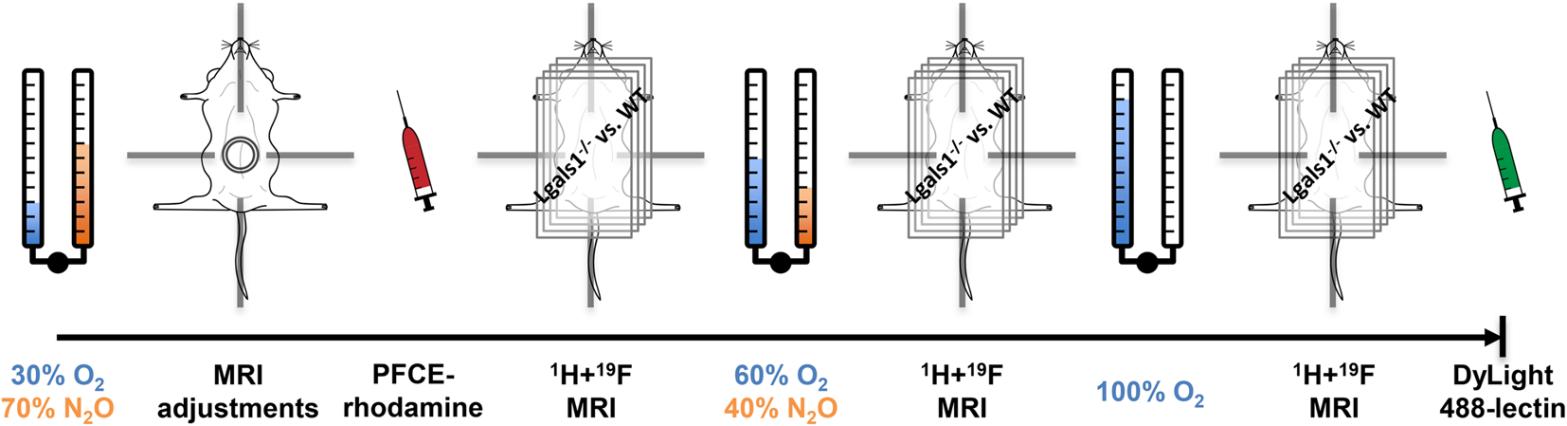
Experimental design. Freely breathing *Lgals1* WT and KO animals underwent serial MRI at different levels of oxygen in breathing air after receiving PFCE emulsion i.v. Fluorophore-coupled lectin was injected at the end of the experiment in order to label the vasculature for histological examinations.

**Figure 2:**
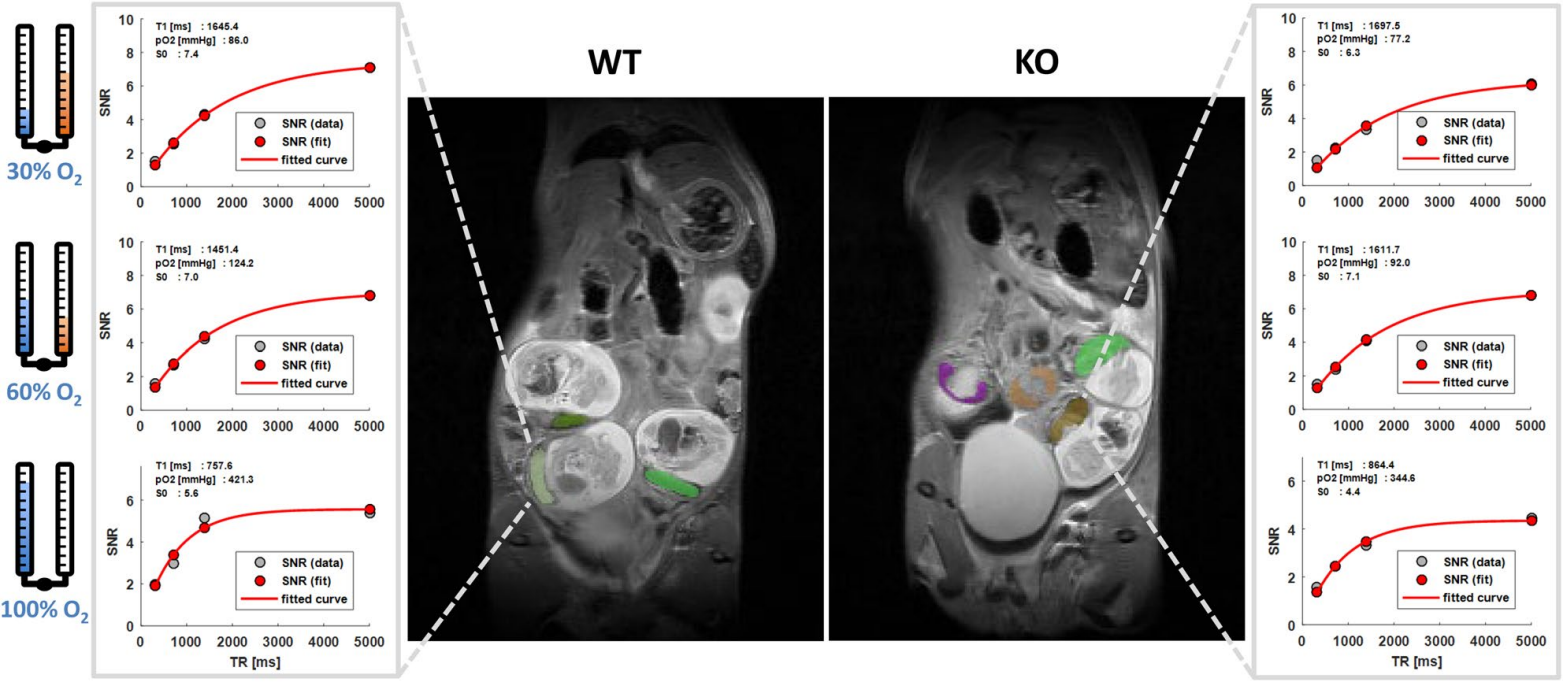
Example data from wildtype (WT) and *Lgals1*^*−*/*−*^ knockout (KO) animal. Colorcoded overlay of placental volumes of interest on anatomical T_2_-weighted MR images. Corresponding mean ^19^F MRI SNR, T_1_ fits and calculated relative ^19^F concentration S_0_ and PO_2_ in mmHg at different levels of oxygen in breathing air are shown for the two placentas marked by dashed lines.

**Figure 3:**
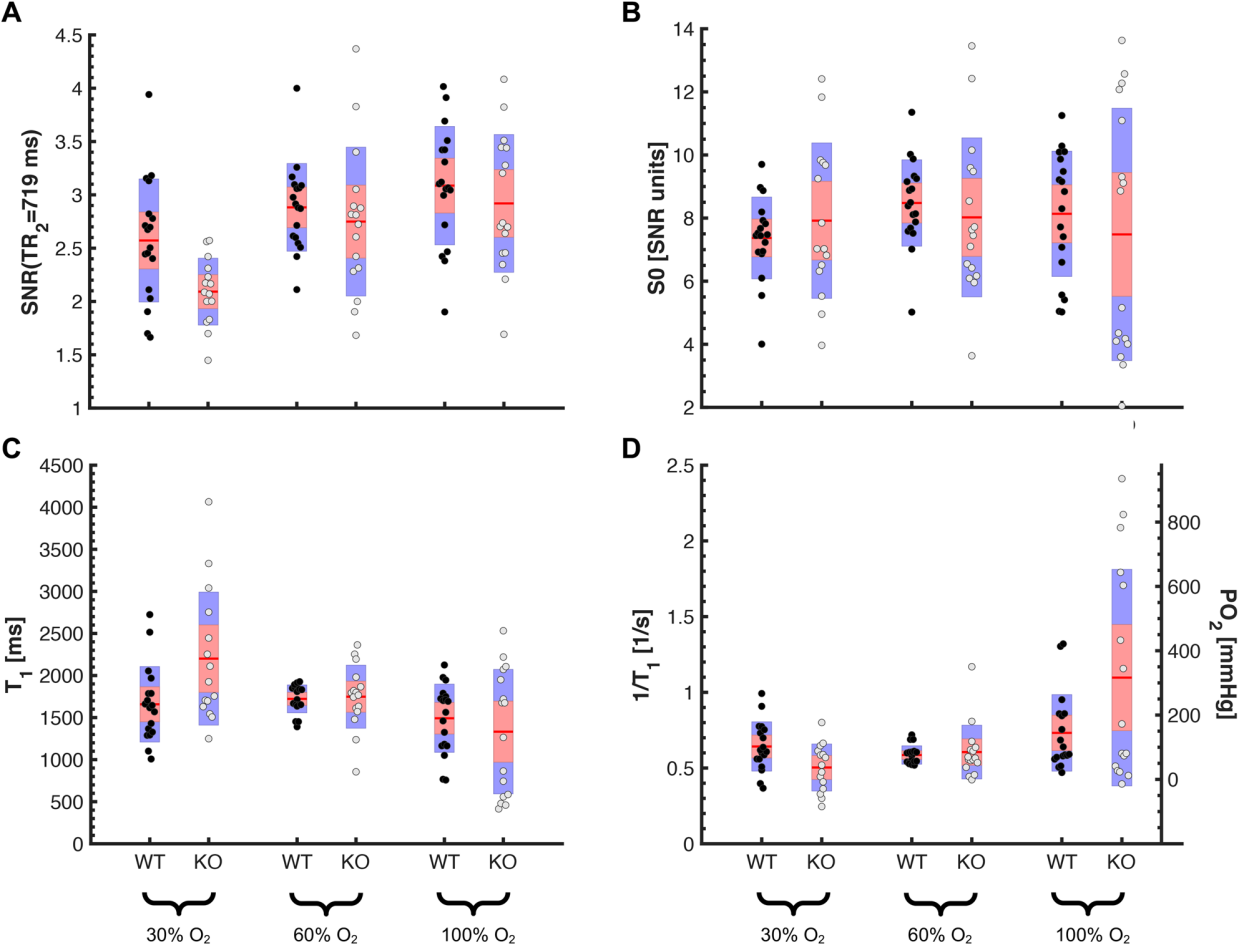
Descriptive statistics of ^19^F MRI parameters in *Lgals1* wildtype (WT, black circles) and knockout (KO, gray circles) placentas at 30%, 60% and 100% oxygen in breathing air. Shown are (**A**) signal to noise ratio on one of the T_1_-weighted images, (**B**) fitted relative ^19^F concentration S_0_, (**C**) fitted T_1_ relaxation time and (**D**) calculated PO_2_ ~ 1/T_1_. Increasing the amount of oxygen in breathing air led to an apparent increase in SNR, decrease in T_1_ and increase in PO_2_ but also an increase in variance. Parameters indicative of decreased oxygenation in knockout animals were only obvious at 30% oxygen. Red lines show mean, red and blue areas correspond to 95% confidence intervals and standard deviation, respectively.

To investigate the effect of increasing levels of O_2_ in breathing gas on measured PO_2_, ^1^H and ^19^F acquisitions were repeated under 60% O_2_ in 40% N_2_O and under 100% O_2_ as illustrated in Fig. 1. Figure 2 shows typical volumes of interest overlaid on anatomical ^1^H images along with T_1_ fits of mean ^19^F signal-to-noise ratio (SNR) and the final result for mean PO_2_ in WT and KO mice at varying oxygen levels in breathing air. The shape of the fitted curves varied substantially, which indicated heterogeneity in PO_2_ between animals, between placentas within one animal and between different breathing conditions. Descriptive statistics showed an expected increase in SNR on T_1_-weighted images (Fig. 3A), a decrease in T_1_ (Fig. 3C) and increase in PO_2_ with increasing oxygen in breathing air, while relative ^19^F concentration S_0_ remained similar. Mixed-effects modeling of SNR(TR_i_, i = 1, 2, 3, 4), T_1_ and PO_2_ between genotype and percent oxygen in breathing air are summarized in Table 1 and confirmed this observation. A significant effect of percent oxygen in breathing air on SNR, T_1_ and PO_2_ was found, even for SNR at the least T_1_-weighting (TR = 5000 ms) indicating an expected strong effect of oxygen level on PO_2_ in the placenta. The fitted equilibrium signal S_0_ = SNR(TR >> T_1_) is a marker of local contrast agent concentration and was neither different between animals nor between breathing conditions.

**Table 1:** Linear mixed-effect modeling including all experimental conditions.

Parameter	Source	Numerator df	Denominator df	F	*p*
PO_2_	Genotype	1	25.938	1.288	.267
	Perc	2	52.556	12.669	3.200E−05
	Genotype × perc	2	52.556	5.524	.007
T_1_	Genotype	1	26.905	.954	.337
	Perc	2	54.419	9.525	2.840E−04
	Genotype × perc	2	54.419	4.602	.014
SNR(TR_1_)	Genotype	1	33.850	4.030	.053
	Perc	2	62.223	24.476	1.440E−8
	Genotype × perc	2	62.223	.830	.441
SNR(TR_2_)	Genotype	1	34.799	2.928	.096
	Perc	2	63.504	18.131	5.900E−7
	Genotype × perc	2	63.504	1.480	.235
SNR(TR_3_)	Genotype	1	33.787	2.510	.122
	Perc	2	62.723	6.718	.002
	Genotype × perc	2	62.723	.042	.959
SNR(TR_4_)	Genotype	1	31.454	.477	.495
	Perc	2	61.012	4.380	.017
	Genotype × perc	2	61.012	.635	.534
S0	Genotype	1	31.086	.055	.816
	Perc	2	60.882	2.369	.102
	Genotype × perc	2	60.882	1.493	.233

Tests of fixed effects (factors genotype and oxygen level) for PO_2_, T_1_, SNR and S_0_. More detailed statistical parameters including estimates can be found in the open data repository.

### ^19^F MRI of placental PO_2_ in preeclampsia mouse model

No effect of genotype was found in any of the parameters in the full statistical linear mixed-effects model but a significant interaction between percent oxygen and genotype at 30% O_2_ in breathing air (Table 1). In line with this, descriptive statistics did not show major genotype differences at higher oxygen levels in breathing air (Fig. 3). We further investigated genotype differences at 30% oxygen by linear mixed-effects modeling (Table 2). A significant effect of genotype was found for PO_2_, T_1_ and the T_1_-weighted images SNR(TR_1_ = 318 ms) and SNR(TR_2_ = 719 ms) whereas this effect vanished for less T_1_-weighting at longer TRs. In *Lgals1*-knockout mice PO_2_ was lower compared to wildtypes (KO: 38 ± 52 mmHg, WT: 102 ± 35 mmHg, data expressed as estimated mean (ME) ± 95% confidence interval (CI), *p* = 0.016) i.e. T_1_ was higher (KO: 2.18 ± 0.43 s, WT: 1.66 ± 0.30 s, ME ± 95%CI, *p* = 0.020). Accordingly, SNR was lower in transgenic animals for T_1_-weighted images at lowest TR (TR = 318 ms, KO: 1.26 ± 0.14, WT: 1.45 ± 0.10, *p* = 0.008) and second-lowest TR (TR = 719 ms, KO: 2.12 ± 0.33, WT: 2.57 ± 0.23, ME ± 95%CI, *p* = 0.009).

**Table 2:** Linear mixed-effect modeling results at 30% oxygen.

Parameter	Source	Numerator df	Denominatordf	F	*p*
PO_2_	Genotype	1	32	6.477	.016
T_1_	Genotype	1	32	6.041	.020
SNR(TR_1_)	Genotype	1	32.000	7.931	.008
SNR(TR_2_)	Genotype	1	32	7.744	.009
SNR(TR_3_)	Genotype	1	32.000	3.616	.066
SNR(TR_4_)	Genotype	1	32.000	.002	.965
S0	Genotype	1	32	.816	.373

Tests of fixed effects (factor genotype) for PO_2_, T_1_, SNR and S_0_. More detailed statistical parameters including estimates and confidence intervals can be found in the open data repository.

### Histology

**Figure 4:**
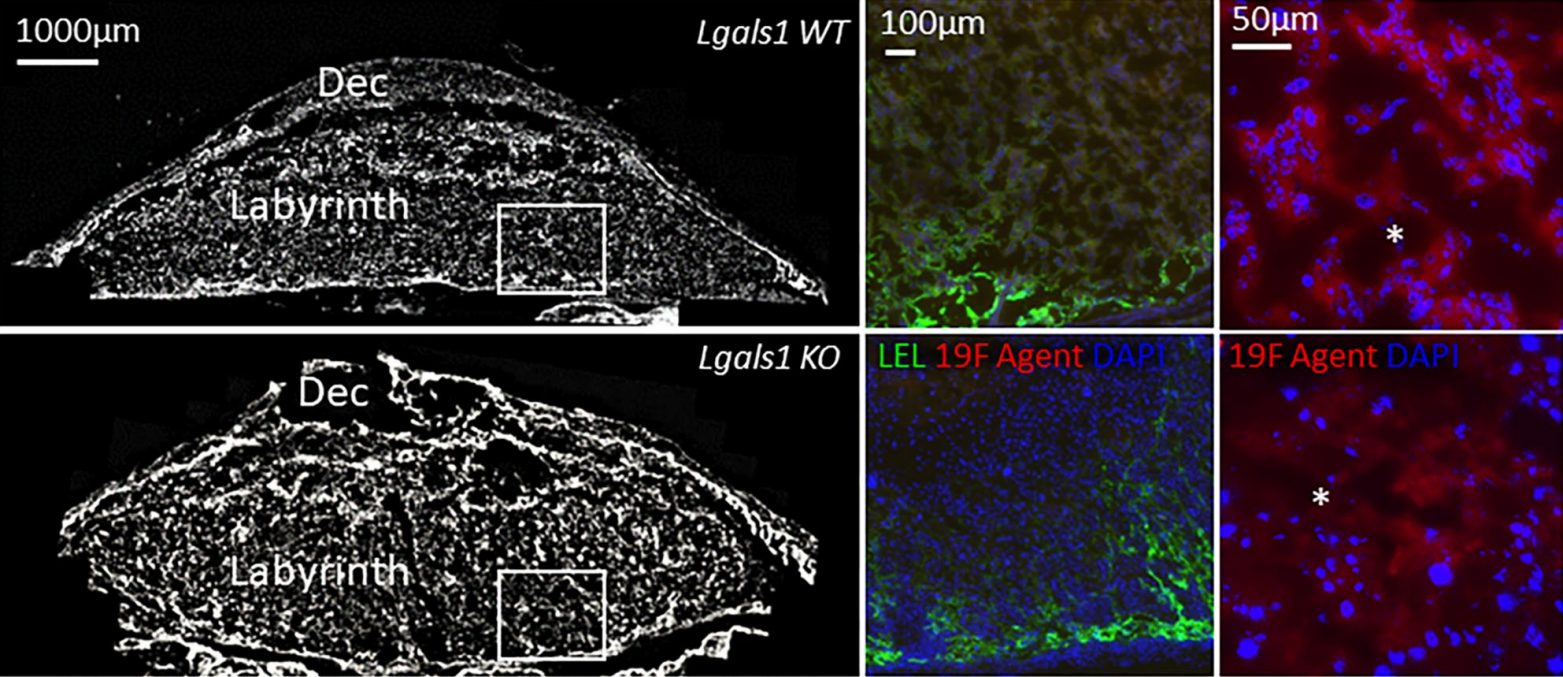
Representative placental histology images derived from *Lgals1*WT (upper) and *Lgals1*KO (lower) showing the decidua (Dec) and placenta layers including the Labyrinth. ^19^F Agent (red) was found in both maternal and fetal blood spaces. Fetal circulation is denoted by the asterisks inserts in both *Lgals1 WT* (upper) and *Lgals1*KO *(*lower). DAPI counterstained nuclei and DyLight 488 labeled Lycopersicon Esculentum (Tomato) Lectin (LEL) was used for imaging vascular structures (endothelial cells). Bars indicate 1000 µm, 100 µm and 50 µm respectively.

In order to qualitatively assess the distribution of ^19^F agent in the maternal and fetal blood pool and in cells of the placenta, fluorescent microscopy of the maternal blood vessels (fluorophore-coupled lectin, green), the ^19^F agent (Rhodamine, red) and cell nuclei (DAPI, blue) was performed. Figure 4, shows the ^19^F agent labeled with Rhodamine crossed the placental barrier and penetrated the maternal and fetal circulation within the labyrinth area of the placenta.

## Discussion

**Figure 5:**
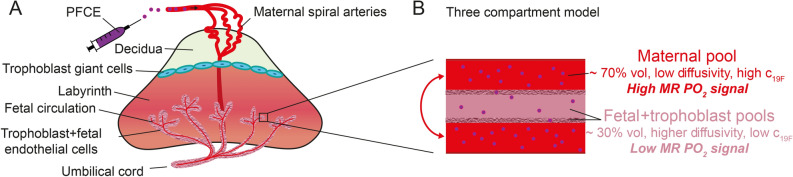
(**A**) Illustration of the mouse placenta and its compartments after i.v. injection of PFCE nanoemulsion. (**B**) Summarizing qualitative and quantitative arguments on volume fraction, diffusivity and ^19^F concentration in a three-compartment model of the labyrinth, the maternal blood pool is the dominating source of MR PO_2_ signal.

So far, most studies on oxygenation ^19^F MRI have focused on tumors and liver. We hypothesized that the placenta is a particularly favorable organ to study with this technique since a large volume fraction consists of maternal plasma which can be reached via simple i.v. injection. In a three compartment model (maternal, fetal and trophoblast pool), a recent diffusion MRI study estimated the maternal blood volume fraction of the placenta at 64.4% (fetal pool: 23.7%, trophoblast pool: 11.9%) and optical imaging confirmed this (~ 70%:30% ratio of maternal:fetal blood pool volume)^13^. In our qualitative histological examinations, we found most fluorescence signal originating from maternal blood but some signal was also present in both trophoblast and fetal pool. Although dissociation of the fluorophore from PFCE nanoemulsion particles is possible^36^, the vesicular patterns of Rhodamine signal hints toward true phenomenon of particles crossing the placental barrier. Mechanisms that regulate the transfer of nanoparticles across the placenta and fetal circulation include simple difussion, active transport, phagocytosis and endocytosis^37,38^. Considering the average PFCE nanoparticle size of 100–200 nm, phagocytosis/endocytosis seem the most likely route. This hypothesis is supported by the finding that caboxylate-modified polystyrene beads with diameters between 20 and 500 nm injected intravenously in pregnant mice accumulated in the placenta via trophoblast uptake^39^. Taken our histological observations together with the quantitative argument on volume fractions, we assume that at least 70% of the contribution to PO_2_ in our study originates from maternal plasma. The contribution is probably higher since diffusivity in the fetal blood pool is orders of magnitude higher than in the other two compartments^13^ which further diminishes the fetal ^19^F MR signal as summarized in Fig. 5. Since PO_2_ is likely to be different for each of the three compartments, ^19^F MRI signal fractions could be estimated via equilibrium signals S0_maternal_, S0_trophoblast_, S0_fetal_ from multiexponential fitting. Adding the assumption of compartmental volume fractions from diffusion imaging could dissect the exact amount of PFCE crossing. Although the ^19^F MR sensitivity in our study was not sufficient to perform such fine-grained models with 6 fitting parameters (S_0_ and T_1_ per compartment), future gains in SNR will enable these types of analyses.

We assumed a steady state of PFCE concentration in the different compartments of the mouse placenta, which is a valid assumption since S_0_, a marker of local ^19^F concentration, was not different between breathing conditions over a period of ~ 1.5 h. During this time, internalization of PFCE nanoparticles by circulating monocytes/macrophages is very likely^40^. There is an oxygen gradient between mitochondria and plasma, which may lead to apparently lower PO2 values of intracellular vs. extracellular PFCE. Using oxygen-sensitive microscopy of green fluorescence protein, intracellular oxygen gradients were found to be very small in monolayer cell culture (~ 0.03 mmHg/µm)^41^ and we thus did not correct for this source of bias.

### Placental PO_2_ under normal and pathological conditions

The most relevant outcome parameter in pathology of placental circulation is the oxygenation of the fetus. We found lower placental pO_2_ in *Lgals1* knockout dams. Due to the low statistical power in our experiments, this finding needs to be confirmed in a hypothesis-driven study. Another limitation of the current study is that most PO_2_ signal originates from maternal pool, i.e. it provides only an indirect marker of fetal oxygenation. A recent optoacoustic study, however, has shown that changes in SO_2_ in the feeding maternal artery, the placenta and the fetus are directly correlated during a gas challenge^17^. The same phenomenon is expected for PO_2_in the range of 0–128 mmHg when PO_2_ and SO_2_ are tightly linked through the oxygen dissociation curve (OCD). Under 30% O_2_, the PO_2_ values found in this study in wildtypes (~ 100 mmHg) are higher than those known from optoacoustics under normoxia with 20% oxygen (SO_2_ ~ 50%, i.e. PO_2_ ~ 30 mmHg using the OCD). The differences cannot be attributed to the higher level of oxygen in breathing air alone. Inaccuracies in the in vitro calibration of R_1_ and PO_2_, discrepancies in anesthesia depth, use of NO_2_ in our study and the inability of optoacoustics to capture dissolved oxygen in blood present further obstacles in direct comparison of results. For hyperoxia (100% O_2_) we show dramatic increases in partial pressure of oxygen (PO_2_ > 200 mmHg) which can only be explained by a significant amount of dissolved oxygen. This observation is in line with other studies of maternal arterial PO_2_ in sow, mare and ewe^42^. In addition, besides the phenomenon of dissolved oxygen in blood, PFCs themselves are able to transport gases and this effect becomes stronger when breathing high levels of oxygen, a phenomenon which is well described in PFC-based therapy of hypoxia^43,44^ and could open the field of placental theranostics.

### Technical considerations: imaging

The first ^19^F MRI experiments date back as early as the beginning of human ^1^H MRI in the 1970s^45^. Paralleling investigations on gas-solving capabilities of PFCs led to the finding of a linear response of ^19^F relaxation rate R_1_ to local oxygen concentration of PFCs^45^. Despite this long history, low sensitivity has limited the widespread use of ^19^F MRI. This becomes particularly severe for oxygenation ^19^F MRI since introducing the necessary T_1_-weighting in images leads to further loss of SNR. Recent technical advances in field strength, MR coil design and PFC emulsion synthesis, however, have boosted the field, particularly with applications in molecular and cellular MRI^25,36^. The experiments of this study were performed at a field strength of 7 T using a double-tuned room temperature 40 mm transmit/receive birdcage resonator coil and the robust turbo spin echo saturation recovery pulse sequence. Within reasonable 20 min imaging time, we managed to acquire sufficient SNR for T1 fitting, albeit at the border of detectability for the highest T_1_-weighting. The SNR was lower compared to in vitro experiments and this could be explained by motion which is omnipresent when imaging the abdomen in vivo. Consequently, our study has several limitations. First, voxel-wise analysis of PO_2_ was not possible due to error propagation in the calculation of PO_2_ from T_1_. Second, smoothing was necessary to improve SNR at loss of resolution. Third, only few T_1_-weighted images were acquired within reasonable time introducing error on the two-parameter fit. These sources of variance and PO_2_-unrelated changes of relaxivity e.g. due to local field distortions at tissue/air interfaces in the bowel, can account for the physiologically impossible values of PO_2_ below 0 or larger 760 mmHg (1 atmosphere) in our data.

Assuming a near-linear relationship of SNR efficiency with field strength (sample-dominant noise), an improvement in sensitivity by factor > 2 is to be expected for the currently strongest MRI system at 21.1 T^46^. Our first results with cryogenic coils for mouse brain ^19^F MRI have shown a similar increase in sensitivity of factor ~ 2 to 3 compared to room temperature coils^21,47^. A transfer of this technology to abdominal imaging in pregnancy is pending but feasible through adaptation of coil geometry. More SNR efficient T_1_ mapping such as Look-Locker methods are generally more prone to artefacts but should further be explored for abdominal ^19^F MRI^48^. Finally, the ^19^F MR signal is limited to maternal blood pool and other, spatially sparse regions of the mother. This fact can be used to accelerate imaging using compressed sensing reconstruction. First studies show that a decrease of imaging time at identical SNR by factor > 2.5 is possible for point-like sources of signal^49,50^. Absence of genotype differences were observed for the two higher levels of oxygen in breathing air, so even in the current study design, a significant improvement in SNR efficiency by factor 3 reduction in scan time can be anticipated when only interested in genotyping. Taken all of these efforts together, an extraordinary gain in SNR efficiency up to an order of magnitude seems possible with modified study designs and technology available already now or in the near future.

### Technical considerations: ^19^F agent

The investigation of PFC emulsions as blood substitutes resulted in toxicological profiling to the level of clinical trials for several compounds such as perfluorooctyl bromide (PFOB) or Perfluorodecalin^25^. However, most of the clinically approved compounds have less favorable properties for oxygenation MRI, e.g. complex spectra with multiple resonances (e.g. PFOB), short biological half lives or complex response of T_1_ to PO_2_ (Perfluorodecalin). Based on a recent comparison of PFCs for preclinical research^51^, we decided to use Perfluoro-15-crown-5-ether since this compound has a strong, narrow single resonance from 20 magnetically equivalent ^19^F nuclei per molecule and a very long half life in blood, allowing long imaging times in a biologically stable state. For oxygenation imaging, the response of PFCE relaxation rate R_1_ to changes in PO_2_ follows a simple linear relationship and the slope is in the medium/upper range compared to other compounds^52^. However, the extremely long half live in the liver^51^ limits the use of this compound to preclinical animal studies. In addition, it needs to be determined if the ^19^F agent has any undesired effect on fetuses. Generally, PFCs are biologically inert and very well tolerated by the body even in high doses. For adults, safety of high doses of i.v. injected PFC emulsions similar to the one used in this study, have been confirmed in clinical studies. Future improvements in SNR, pulse sequences for complex spectra^53^ and shortening of imaging times will enable the use of clinically more favorable PFCs such as PFOB, which has a similarly strong 1/T_1_ to PO_2_ response for the CF_3_ group (unpublished in vitro data). Further improvement include the use of higher concentrated emulsions in order to increase SNR and PEGylation of particles to prolong systemic circulation time^54^.

## Conclusion

Combining advances in small animal MRI hardware and ^19^F agent synthesis, this study presents the first important step in using ^19^F MRI for placental phenotyping. Detection of decreased PO_2_ in *Lgals1*deficient mice highlights the potential of the technique in a mouse model of preeclampsia, one of the most detrimental pregnancy complications. We foresee two important use cases of placental oxygenation ^19^F MRI. First, it presents a screening method in animal models of placental malfunction, especially when oxygenation is a primary outcome measure and is uncoupled from surrogate markers such as perfusion or blood oxygenation SO_2_. Second, since ^19^F MRI provides absolute values of PO_2_, it bears potential to calibrate less invasive techniques based on conventional ^1^H measurements such as BOLD/TOLD MRI^55^. This is the first study to characterize placental oxygenation in vivo during uneventful pregnancy and a pathological disorder such as preeclampsia using ^19^F MRI.

## Methods

### Animals

Inbred 129/P3J lectin, galactoside-binding, soluble, 1 (*Lgals1*) wildtype (WT) and deficient (KO) mice were maintained in our animal facility with a 12L/12D cycle^35^. Eight- to 10-week-old virgin female *Lgals1* WT or KO mice were mated with 8- to 14-week-old *Lgals1* WT or KO males respectively. Females were inspected daily for vaginal plugs; sighting a vaginal plug was designated as day 0 of pregnancy. Pregnant *Lgals1* WT (normal pregnancy, n_m_ = 3 mothers/n_p_ = 19 placentas) or KO (preeclampsia model, n_m_ = 4/n_p_ = 17) female mice were subjected to ^19^F MRI on E14/E15 and the whole implantation units were harvested to investigate the presence of contrast dye in the placenta circulation. Animals were scanned in order of breeding, no additional randomization was performed. All experimental protocols were approved by Charité and the state authority for Animal Use in Research and Education Berlin committee under license G0286/16.

### Perfluorocarbon emulsion

PFCE emulsions were prepared using 2.4% (w/w) phospholipid (Lipoid E80S, Lipoid AG, Ludwigshafen, Germany), 0.5 mol% Rhodamine-DHPE (Lissamine Rhodamine B 1,2-Dihexadecanoyl-sn-Glycero-3-Phosphoethanolamine, Triethylammonium Salt, Thermo Fisher Scientific, Waltham, MA, USA) dissolved in 10 mM phosphate buffer (7 mM Na_2_HPO_4_, 3 mM NaH_2_PO_4_, pH 7.4 isotonized with 2.5% (w/w) glycerol) and mixed for 30 min. Next, 40% (w/w) PFCE (ABCR, Karlsruhe, Germany) was added to the dispersion and a crude emulsion was formed by high shear mixing (Ultra Turrax TP 18/10; IKA-Werke, Staufen, Germany). Finally, high shear homogenization was performed in 10 cycles at 1000 bar using a LV1 microfluidizer (Microfluidics Corp, Westwood, MA, USA).

### MRI

MRI was performed at 7 T on a Bruker BioSpec 70/20 (Bruker BioSpin, Ettlingen, Germany) with Paravision 6.0.1 software using a double-tuned ^1^H/^19^F 40 mm diameter transmit/receive volume coil (Bruker). Anesthesia was initiated with 2–3% isoflurane and maintained using 1.5–2% isoflurane in a 30%/70% mixture of O_2_/N_2_O. To allow i.v. injections during MRI, a catheter was surgically placed in the femoral vein and the animal was positioned in supine position on an animal cradle. The head was placed in the anesthesia mask with a toothbar. Extremities were gently fixed with adhesive tape to stretch the animal and reduce abdominal motion. An MRI-compatible small animal monitoring system was used to monitor respiration and temperature (SA instruments, Stony Brook, NY, USA). Temperature was maintained at 37 ± 0.5 °C using a rectal temperature probe linked to a warm-water-driven feedback-controlled blanket. Respiration was monitored with a pressure-sensitive pad placed underneath the back of the animal. T_2_-weighted anatomical images were acquired using a ^1^H 2D respiration-triggered rapid acquisition with relaxation enhancement (RARE) sequence with field-of-view (FOV) = 39.2 mm × 51.2 mm, image matrix (MTX) = 196 × 256, slice thickness 1 mm, 20 coronal slices with 0.2 mm gap between adjacent slices, fat suppression, repetition time (TR) = 1.6 s, RARE factor = 8, echo time distance (ΔTE) = 8 ms, effective echo time (TE) = 16 ms, readout bandwidth (BW) = 50 kHz, number of averages (NA) = 6, time of acquisition (TA) = 3:50 min, effective TA including dead time due to triggering = 5–7 min. 400 µL PFCE emulsion were injected very slowly i.v. over 10 min and the system was switched to ^19^F. The basic frequency was calibrated to the PFCE frequency at around − 26 kHz from the scanner software standard ^19^F frequency using a free induction decay experiment (SINGLEPULSE, bandwidth BW = 3 kHz, TR = 110 ms, NA = 500, TA = 55 s). The reference pulse gain was copied from the ^1^H acquisition since this parameter was similar for ^1^H and ^19^F channels of the double-tuned birdcage coil as tested in a separate in vitro experiment on a tube with the pure PFCE emulsion. ^19^F T_1_ weighted images were acquired using a non-triggered, saturation recovery 2D RARE pulse sequence (FOV = 39.2 mmx51.2 mm, MTX = 64 × 84, 8 coronal slices with 0.4 mm gap, 2 mm slice thickness, multiple TRs of TR_1_ = 318 ms/TR_2_ = 719 ms/TR_3_ = 1398 ms/TR_4_ = 5000 ms, RARE factor = 2, ΔTE = TE = 14.3 ms, BW = 10 kHz, NA = 5, TA = 19:50 min). A narrow excitation and refocusing pulse bandwidth (1 kHz) was chosen to avoid chemical shift artefacts from isoflurane signal. In order to transfer anatomical volumes of interest (VOI) from the anatomical ^1^H scan to the ^19^F T_1_ weighted images, the center and orientation of both slice stacks were identical.

Each time after changing breathing gas O_2_ levels, we allowed > 10 min until the center of k-space of the ^19^F scans in order to ensure an equilibrium of blood gases including a 5 min resting period and the 5–7 min during ^1^H MRI acquisition (Fig. 1).

### MRI PO_2_ analysis

MRI analysis was performed blinded to the genotype of animals. To reduce the impact of noise, T_1_ weighted images were preprocessed in ImageJ (v1.52a, https://imagej.nih.gov) using the following steps:

1. Voxel-wise conversion of signal intensity into SNR. In order to mitigate impact of non-Gaussian noise distribution in low SNR magnitude images, we used the approximation by Gudbjartsson and Patz^56^
  $$SNR = {\sqrt {\left( {SI_{m}^{2} -\sigma_{g}^{2} } \right)}} \bigg / {\sigma_{g}}$$
  with magnitude signal intensity on the original image *SI*_*m*_. The noise in magnitude images *σ*_*m*_ was measured as the standard deviation of *SI*_*m*_ in a region of interest with no signal and Gaussian noise was calculated via
  $$\sigma_{g} = \frac{{\sigma_{m} }}{\sqrt{2 - \frac{\pi }{2}}}$$
2. Bicubic linear interpolation to the ^1^H image resolution
3. Gaussian blur filtering using 3 pixel kernel width
4. Exclusion of all voxels with an SNR < 5 on the TR = 5 s image from all further analysis

Volumes of interest of each placenta were drawn on ^1^H anatomical MR images using ITK Snap (v. 3.6.0, http://www.itksnap.org)^57^ and transferred to the preprocessed ^19^F SNR maps. Mean ^19^F SNR was measured for each TR and exported into MATLAB (version R2014b, MathWorks, Natick, USA). The relaxation time T_1_ of the placenta was calculated by fitting the saturation recovery signal model

$$SNR = S_{0} \left( {1 - e^{{ - \frac{TR}{{T_{1} }}}} } \right)$$

The fitted equilibrium signal S_0_ is proportional to the ^19^F concentration and was analyzed separately. PO_2_ was calculated using the in vitro calibration of a previous study^21^

$$PO_{2} \left[ {{\text{mmHg}}} \right] = {\raise0.7ex\hbox{${470.81}$} \!\mathord{\left/ {\vphantom {{470.81} {T_{1} \left[ {\text{s}} \right]}}}\right.\kern-\nulldelimiterspace} \!\lower0.7ex\hbox{${T_{1} \left[ {\text{s}} \right]}$}} - 200.14$$

### Histology

At the end of the MRI acquisition, pregnant mice received 100 µL DyLight 488 Labeled LycopersiconEsculentum (Tomato) Lectin (LEL, TL, Vector Laboratories, BiozolDiagnostica) dissolved in 100 µL PBS i.v. over 5 min. Animals were euthanized by cervical dislocation. The entire pregnant uterine horns were dissected and first rinsed in 0.1 M PBS, cryoprotected in Tissue-Tek (VWR), frozen and kept at − 80 °C until processing. Serial cryosections from whole implantations on E14/E15 were cut at 8 μm. The slides were washed in TBS for 5 min and nuclei in all sections were counterstained by incubating 5 min in DAPI solution, followed by washing and mounting in ProLong Gold (Invitrogen, Thermo Scientific; 99-904-02). Sections were analyzed using a Pannoramic Digital Slide Scanners MIDI microscope (3DHistech).

### Statistics

Mean SNR(TR_i_) (i = 1, 2, 3, 4), i.e. one value for each of the four preprocessed ^19^FT_1_-weighted images, T_1_ relaxation time, PO_2_ and S_0_ in the placenta were statistically evaluated in SPSS (v. 25.0., IBM Corp., Armonk/NY, USA). Mothers were assumed to be the statistically independent unit, i.e. the analysis of placentas was corrected for nesting. Linear mixed-effects modeling (SPSS MIXED) was used with genotype as the between factor and percent oxygen in breathing air as a within factor. Fixed effects of genotype, percent oxygen and their interaction were analyzed. Significant interaction effects were further investigated for an effect of genotype using post-hoc linear mixed model for each level of percent oxygen. Significant effects were further investigated using unpaired two-tailed t-tests. Each parameter of interest was treated independently without post-hoc correction between parameters.

## Data Availability

Work instructions, MRI raw data, processed data used for calculating PO_2_ and detailed statistical output can be found on https://doi.org/10.5281/zenodo.3876271.
